# In-vitro comparison of LC-DCP- and LCP-constructs in the femur of newborn calves – a pilot study

**DOI:** 10.1186/1746-6148-8-139

**Published:** 2012-08-21

**Authors:** Mona Hoerdemann, Philippe Gédet, Steven J Ferguson, Carola Sauter-Louis, Karl Nuss

**Affiliations:** 1 Clinic for Ruminants, University of Munich, Munich, Germany; 2 Institute for Surgical Technology and Biomechanics, University of Berne, Berne, Switzerland; 3 Department of Farm Animals, University of Zürich, Zürich, Switzerland

## Abstract

**Background:**

To compare the biomechanical in-vitro characteristics of limited-contact dynamic compression plate (LC-DCP) and locking compression plate (LCP) constructs in an osteotomy gap model of femoral fracture in neonatal calves. Pairs of intact femurs from 10 calves that had died for reasons unrelated to the study were tested. A 7-hole LC-DCP with six 4.5 mm cortical screws was used in one femur and a 7-hole LCP with four 5.0 mm locking and two 4.5 mm cortical screws was used in the corresponding femur. The constructs were tested to failure by cyclic compression at a speed of 2 mm/s within six increasing force levels.

**Results:**

The bone-thread interface was stripped in 21 of 80 cortical screws (26.3%) before a pre-set insertion torque of 3 Nm was achieved. Only 3 corresponding intact pairs of constructs could be statistically compared for relative structural stiffness, actuator excursion and width of the osteotomy gap. Relative structural stiffness was significantly greater, actuator excursion and width of the osteotomy gap were significantly smaller in the LCP constructs. While failure occurred by loosening of the screws in the LC-DCP constructs, locking constructs failed by cutting large holes in the soft distal metaphyseal bone.

**Conclusions:**

An insertion torque sufficient to provide adequate stability in femurs of newborn calves could not be achieved reliably with 4.5 mm cortical screws. Another limiting factor for both constructs was the weak cancellous bone of the distal fracture fragment. LCP constructs were significantly more resistant to compression than LC-DCP constructs.

## Background

Fractures of the os femoris are common in newborn calves [1-5]. Femoral and tibial fractures rank second to metacarpal and metatarsal fractures in order of frequency of long bone fractures in cattle [6,7]. The most common cause of femoral fractures in calves is excessive traction during delivery, but trauma, such as the dam standing on the calf, and bovine viral diarrhoea (BVD) virus infection are other causes [8,9].

**Figure 1:**
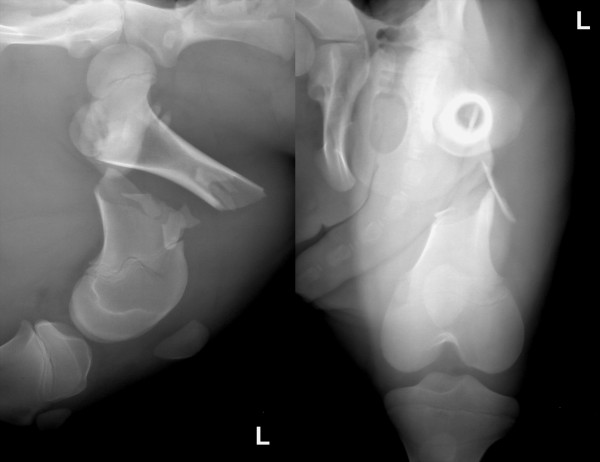
**One-day-old Simmental bull calf, mediolateral and craniocaudal radiographic views of the most common femoral fracture type.** The fracture is irregular oblique at the transition from the diaphysis to the distal metaphysis. There is severe displacement and overriding of the main fragments and comminution at the fracture site.

In calves, femoral fractures occur most often in the proximal epiphysis and distal metaphysis [1,2,10,11]. In a study of newborn calves, 28 of 50 femoral fractures (56%) were located in the distal metaphysis [2]. Because the cortex becomes considerably thinner at the transition from the diaphysis to the metaphysis, this part of the femur has only limited axial strength [12]. If a “stifle lock” occurs during delivery of a calf in anterior presentation and dorsopubic position, forced extraction can lead to wedging of the femur, which increases axial loading, thus leading to femoral fracture [12]. Excessive traction on the hind limbs of a calf in posterior presentation may result in femoral fractures if a lever action is applied on the hind limbs [2]. Most femoral fractures in newborn calves are irregular transverse or oblique fractures. Gross displacement of the fragments (Figure 1), extensive stripping of the periosteum and injury to adjacent tissues are typically seen [2,3,13-15].

Conservative treatment of femoral fractures is rarely successful [2-4,15]. When a Thomas splint [4] or intramedullary pins [3] were used for fixation, the outcome was significantly worse for fractures in the distal metaphysis compared with fractures of the diaphysis. Fixation of distal metaphyseal femoral fractures using Steinmann pins was associated with pin migration and instability [3,5,11]. In contrast, the outcome after plate osteosynthesis was not affected by the location of the fracture [2,16]. Recently, a novel intramedullary interlocking nail was used for repair of femoral fractures in newborn calves with a good prognosis regardless of the location of the fracture [5].

The softness of femoral bone is one of the major concerns of plate osteosynthesis in neonatal calves [1,2,13]. Soft neonatal bone can predispose to loosening of the screws and subsequent instability of the fixation. In human medicine, angle-stable implants are used for osteosynthesis in soft and osteoporotic bones. The aim of the present in-vitro study was to compare a limited-contact dynamic compression plate (LC-DCP) construct and a locking compression plate (LCP) construct in an osteotomy-gap model of femoral fractures in newborn calves.

## Methods

**Figure 2:**
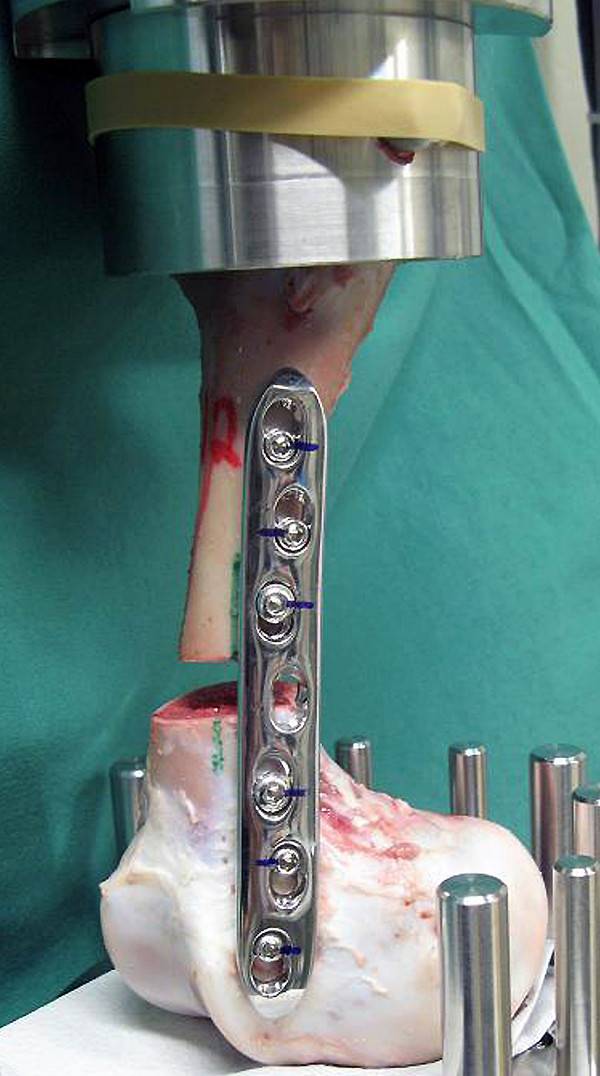
**Coupling of a LCP construct to the testing machine. The femoral head has been truncated to fit into the loading frame.** The axial load is applied through the diaphysis to the distal fragment. Lines drawn close to the plate and from the screw heads to the plate allow assessment of plate and screw displacement.

Fourteen pairs of intact femurs were collected from calves that had died or were euthanized for other reasons within the first 10 days of life. Of these, four pairs were used for basic studies to exactly determine the shape and location of the epiphyseal lines and for the way the femurs were coupled in the testing machine (Figure 2). Compression loading was achieved by axial excursion of the actuator of the machine at a speed of 2 mm/s. In addition, the position and length of the plates and the insertion torque were predetermined in one pair of femurs. The insertion torque was set at 3 Nm for the cortical screws and at 4 Nm for the locking screws.

**Figure 3:**
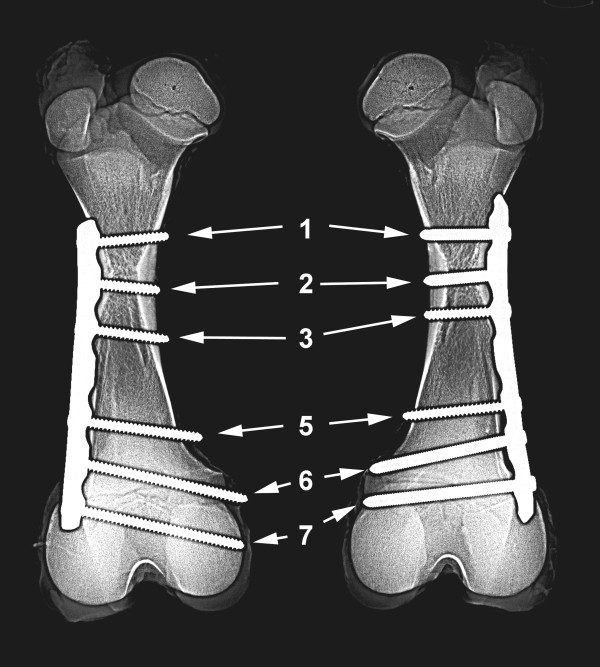
**Craniocaudal radiographic view of the femurs obtained at postmortem from a five-day-old Simmental bull calf.** A broad 7-hole LC-DCP (left) has been used in one femur and a 7-hole LCP (right) was used in the other femur. The plates are placed craniolaterally on the bones and as far distally as possible. The plate holes and screws are numbered from proximal (No. 1) to distal (No. 7). In the LCP, two central 4.5 mm cortical screws and 5.0 mm locking screws are used.

Ten bone pairs from nine Simmental and one Holstein-Friesian calf were used for the pilot study. Three calves were female and seven were male. The median age was six days (range 1–10 days) and the median bodyweight was 41.3 kg (range 36.6 - 50.0 kg). Radiographs of each bone pair were taken to rule out abnormalities such as BVD virus-induced changes, and after the plates had been fixed to the bones to document the location of the plate and screws. The plate holes and screws were numbered from proximal (No. 1) to distal (No. 7) (Figure 3).

Before creation of the bone defect, a broad 7-hole LC-DCP was used in one femur and a 7-hole LCP was used in the contralateral femur of the same animal. The plates were contoured to the craniolateral surface of the bones. They were placed as far distally as possible adjacent to the femoropatellar joint pouch. Six 4.5 cortical screws were inserted with the LC-DCP plate. For the LCP, two 4.5 mm cortical screws were placed closest to the defect, and 5.0 mm locking screws were used in the four remaining peripheral holes. The screws were not tightened to the pre-set final torque. In each bone of every pair, a 12-mm osteotomy defect was created with an oscillating saw at the distal aspect of the femoral diaphysis. The screws were finally tightened using a screw driver with adjustable torque (torque screw driver, Hoffmann Group, Munich, Germany). Lines were drawn with a waterproof marker from the screw heads to the plate surface and additional lines were drawn on the bones along the border of the plates. These were used to identify rotation and migration of the screws in the plate holes and displacement of the plate. The bone-plate constructs were mounted in a servo-hydraulic testing machine (Bionix 858, MTS Systems, Minneapolis, USA; Figure 2) and tested at axial compression using 100 loading-relaxation cycles per testing level. The maximum force was increased after each testing level, from 500 N in the first stage to 1000 N in the second stage followed by four subsequent steps of 250 N each to a maximum force of 2000 N. The axial excursion of the actuator of the testing machine was recorded at cycle 1, 50 and 100 during each level. The number of test cycles to failure was recorded for each construct. Failure was defined as bone-to-bone contact between fragments. After failure, the constructs were examined and the plates removed to record the extent of drill-hole deformation and the number of permanently deformed screws.

For descriptive data analysis of all constructs, SPSS® 16.0 (SPSS Inc., Illinois, Chicago, USA) and Microsoft® Office Excel 2003 (Microsoft Corporation, Redmond, Washington, USA) were used. Frequencies were compared with Chi-squared tests or Fisher’s exact tests for differences between the two constructs. SAS® 9.1 (SAS Institute Inc., Cary, North Carolina, USA) was used to correct the data for repeated measures within a bone pair, and the animal from which the bones originated was defined as a random effect (PROC Mixed procedure). P ≤ 0.05 was considered statistically significant.

## Results

Stripping of the bone threads of the screw hole before the predetermined torque of 3 Nm had been reached occurred during insertion of 21 of 80 (26.3%) cortical screws; 19 of these were located in the LC-DCP and 2 in the LCP constructs (p < 0.001). Tightening of all screws of a construct with the defined torque was only achieved in three LC-DCP and eight LCP constructs (p = 0.07; Fisher’s exact test). Only the three intact corresponding constructs in which the screws could be tightened to the pre-set torque were statistically analysed with respect to relative structural stiffness, maximum axial excursion and width of the osteotomy gap. However, all 20 constructs were tested until failure and then compared with descriptive statistics.

**Figure 4:**
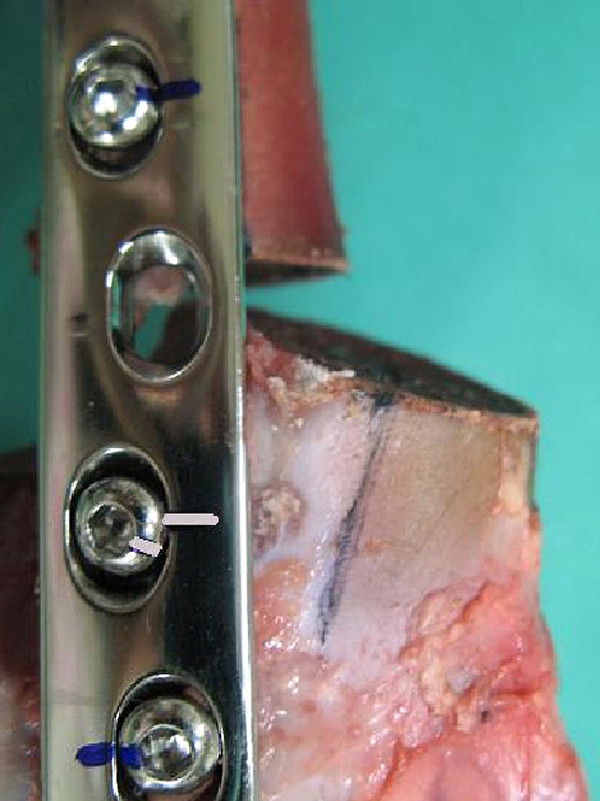
**LC-DCP construct after the 4th testing stage, close-up.** The distal bone segment is tilted cranially and the osteotomy gap is increased cranially and decreased caudally. Loosening of screw No.5 is evident because of deviation of marks on the plate and screw that were in line at the start of testing.

After the first testing stage, there was further loosening of 18 of the remaining 41 (43.9%) cortical screws in the LC-DCP constructs. In the LCP constructs, eight of the remaining 58 (13.8%) screws, five cortical and three locking screws, loosened (difference between the two constructs: p = 0.001; Figure 4). In the LC-DCP constructs, all the screws in the two most distal positions (screws No. 6 and 7) were loose after the second testing level (1000 N).

**Figure 5:**
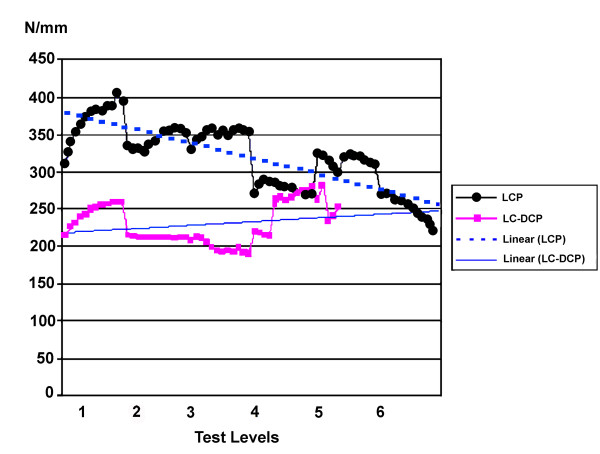
**Changes in relative structural stiffness (median of each group of 3 femora) during subsequent test levels.** Stiffness of LCP constructs is significantly higher in the first 4 test levels and decreases linearly. The linear increase in the LC-DCP constructs possibly is a result from shear forces which occurred after screw loosening and slowed down extraction of the screws. The LC-DCP group failed at the 5th test level.

Relative structural stiffness of the three corresponding intact LCP constructs was significantly (p < 0.001) greater than that of the LC-DCP constructs during the first three loading levels and also during the 4^th^ loading level (p < 0.05; Figure 5). All three LC-DCP constructs failed before the 38th cycle of the 5th test level. Failure of the three LCP constructs occurred at least one test stage later than the corresponding LC-DCP constructs. The maximum axial excursion of the head of the testing machine during compression in the first four stages was significantly smaller in the three LCP constructs than in the three LC-DCP constructs (p < 0.001; Table 1). Likewise, the changes in the width of the osteotomy gap were significantly smaller in the three LCP constructs (p < 0.001). Both groups showed a progressive increase in the maximum axial excursion (Table 2).

**Table 1:** Maximum excursion (in mm, median values for each construct) of the actuator for test cycle 5, 50 and 100

**Test level**	**Loading cycle 5**		**Loading cycle 50**		**Loading cycle 100**	
	**LCP**	**LC-DCP**	**LCP**	**LC-DCP**	**LCP**	**LC-DCP**
**1**	1,79	2,61	2,53	3,75	2,83	4,11
**2**	3,45	5,04	4,65	6,59	4,98	7,07
**3**	3,88	6,20	4,92	8,46	5,41	9,42
**4**	5,68	7,06	7,34	9,0	8,18	11,47
**5**	5,73	7,36	7,38		8,42	
**6**	7,90		10,59			

At the beginning of each test level, the starting point of the actuator was set to zero.

- = failure of the construct.

**Table 2:** Maximal changes of gap width (calculated from the median values) after the first 4 test levels

**Measurement point**	**Maximum width change (in percent)**	
	**LCP**	**LC-DCP**
**1**	1,68%	16,87%
**2**	1,77%	11,55%
**3**	19,54%	43,75%
**4**	11,31%	35,68%

The axial compression testing led to craniolateral rotational movement (Figure 2) of the distal fragment in all the constructs. This deviation in the axis appeared early in the testing cycles and was more pronounced in the LC-DCP constructs. Screw No. 6 appeared to be the centre of this rotational movement based on the observation that there were only minor changes in the appearance of screw hole No. 6, and more severe changes at screw holes No. 5 and 7. The drill holes did not have major macroscopic changes in the trans-cortex and proximal fragment after removal of all constructs. In the cis-cortex of the distal fragment, the drill holes were markedly widened, mostly in a horizontal direction.

During the experiment, 32 of 120 (26.6%) screws underwent permanent plastic deformation under loading; all screws were from the distal fracture fragment. In total, 25 of the 80 cortical (31.3%) and seven of the 40 locking screws (17.5%) were affected (p = 0.108). The latter were deformed at the transition from screw head to body, while the cortical screws were deformed at the transition from screw head to body or in the middle of the body. Cortical screw No. 5 underwent permanent plastic deformation in seven of 10 constructs in both groups.

## Discussion

To the authors’ knowledge, there are no published guidelines regarding the optimum insertion torque for cortical screws in the calf femur. Although a preliminary trial established an insertion torque of 3 Nm for 4.5 mm cortical screws, a large number of cortical screws caused stripping of the thread in the soft bone of the distal metaphysis and epiphysis before the pre-set value was reached. This discrepancy is believed to mainly reflect the relative weakness of calf bones [17] and to a much lesser extent the differences in the structures of the femoral bones among individual calves. An insertion torque greater than 3 Nm is necessary to achieve adequate stability and to prevent movement between the components of an osteosynthesis [18,19]. The highest torque achievable in osteoporotic bone is generally considered to be 3 Nm [18,20-22]. It can therefore be concluded that the femoral metaphyses of newborn calves are as weak as osteoporotic bone. As a consequence of this weakness, no cortical screw in the distal fragment of the LC-DCP group remained firmly fixed after the second testing level. The number of loosened screws was much higher in the LC-DCP constructs which was associated with earlier implant failure. In clinical cases, stripped cortical screws are commonly replaced with 6.5 mm cancellous screws [2,18]. However, cortical screws were chosen in the present study because cancellous screws did not increase the fixation strength of an LC-DCP construct in an osteoporosis model [23,24]. In addition, in a study that investigated the holding power of different screws, there were no significant differences among the holding power of 4.5 mm and 5.5 mm cortical screws and 6.5 mm cancellous screws in the diaphysis and metaphysis of calf femurs [25]. The insertion torque of cancellous screws in the metaphysis and epiphysis of calf femurs should be investigated in further studies.

The transition from the diaphysis to the distal metaphysis of the femur was chosen for the experimental fracture site because this is a common location of fractures in newborn calves. Furthermore, plate fixation at this location was thought to be more suitable than fixation using an intramedullary pin, whereas in the diaphysis either technique may be used [3,11]. A relatively large osteotomy gap of 12 mm was chosen to ensure that the plate, rather than the fracture fragments, was the load-transferring component of the osteosynthesis [21,26-28]. Such a large osteotomy gap is not usually seen in clinical cases and was probably a main factor in the rapid screw loosening in the LC-DCP group of the present study.

Permanent plastic deformation occurred in almost twice as many cortical screws than locking screws, which underlines the greater strength of the locking screws. In the locking screws, bending occurred more commonly at the transition of the body to the head of the screw. In contrast, bending in cortical screws occurred at the head or at the body. Interestingly, the proportions of bent screws in the No. 5 position were the same for LCP and LC-DCP constructs after failure. This means that the peripherally-placed locking screws could protect the cortical screw in the more central position only during the initial testing stages. Conventional cancellous screws at the plate ends may be considered in an attempt to reduce stress concentration [29] in calf femurs.

The initial load of 500 N corresponds to a weight of approximately 50 kg, which corresponds to the weight of a newborn calf. However, it can be assumed that the limbs of a newborn calf that struggles to stand for the first time undergo loads that correspond to multiples of their bodyweight. In a study involving human femurs, a load corresponding to three times the bodyweight was used to test different fixation systems [30].

Relative structural stiffness was significantly greater in the LCP constructs than in the LC-DCP constructs. Likewise, maximum axial excursion and width of the osteotomy gap were significantly smaller in LCP in a validated model of the osteoporotic femoral diaphysis [24].

**Figure 6:**
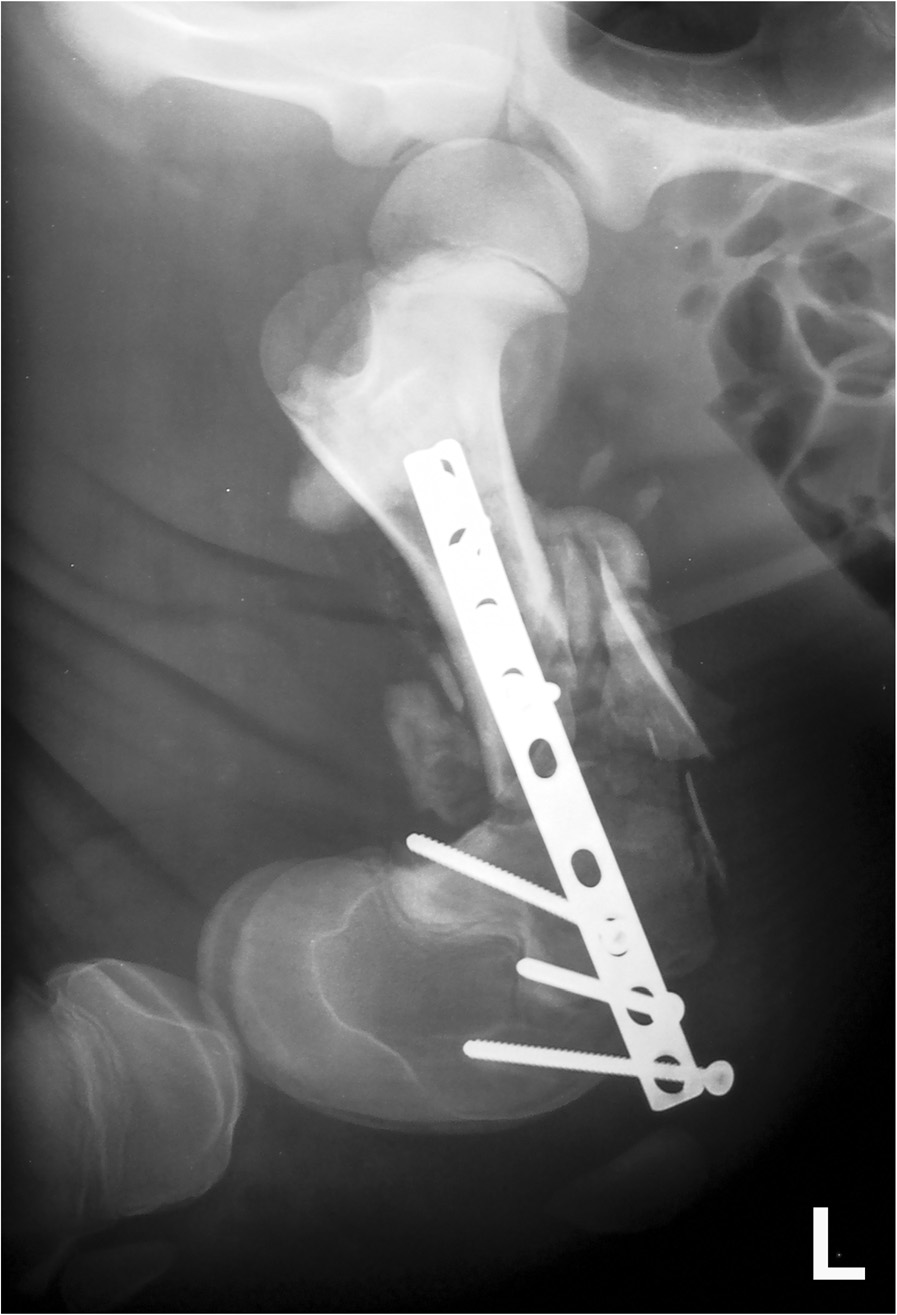
**Mediolateral radiographic view of a failed plate osteosynthesis of the left femur of a 3-week-old male Simmental calf 3 weeks after surgery.** The 4.5 mm cortical screws of the distal fracture segment became loose and the fracture site collapsed.

The rotational deviation of the distal fracture fragment in all the constructs was most likely the result of the eccentric orientation of the plate relative to the concavity of the femoral condyles (Figure 2). Thus, although this was not intentional, rotational stability was also tested in addition to axial stability. The deflection of the distal fragment, which has also been observed in clinical cases (Figure 6), underlined that the area of bone-screw interface of the distal fragment was the weakest element of the fixation [24,31]. Bicortical screws were used exclusively in the present study because they are recommended for osteosynthesis in weak or compromised bones [20]. The deflection resulted in a rotational movement of the screw heads in the cis-cortex, while the apex of the screws was still fixed in the trans-cortex. This finding supports the idea of using far cortical locking screws [27], which would help to reduce damage to the cis-cortex.

The distal position of the plate caused bridging of the growth plate by the two most distal screws. This was inevitable in the LCP because of the predetermined entry angle of the locking screws, whereas it could have been partially prevented in the LC-DCP constructs by changing the entry angle of the cortical screws. However, for better comparison of the two constructs, similar insertion angles were used in all screws. Bridging of the growth plates by screws should be avoided in clinical cases, but in femoral fractures close to the distal metaphysis this is not always possible without severely compromising stability in large animals. In such cases, the implants should be removed as soon as possible once healing of the fracture is complete [2,19,32].

In large animal long-bone fractures, the use of two plates in a 90-degree configuration is recommended [33]. In an experimental study that compared different implants, osteosynthesis with two LCPs was better than osteosynthesis with two DCPs, LC-DCPs or clamp rod internal fixation systems for the fixation of a simple oblique fracture in an equine long-bone model [34]. Because the interaction of two plates would have complicated the interpretation of the findings, we chose to use only one plate in our model. However, in a clinical situation, a second plate could have prevented the rotational deviation.

## Conclusions

An insertion torque sufficient to provide adequate stability for plate fixation in femurs of newborn calves could not be achieved reliably with 4.5 mm cortical screws. Femoral metaphyses of the calves studied were as weak as human osteoporotic bone. Cortical screws in the metaphyses should be replaced by cancellous screws in further studies. A main limiting factor for the stability of both LC-DCP and LCP constructs was the distal fracture fragment. However, relative structural stiffness was significantly greater and actuator excursion and width of the osteotomy gap significantly smaller in the LCP constructs.

## Authors' contributions

All authors were involved in the study design. Mona Hoerdemann carried out the experimental studies together with Philippe Gédet and was assisted by Karl Nuss and Steven Ferguson. Mona Hördemann and Carola Sauter-Louis completed the statistical analysis, and the manuscript was written by Karl Nuss. All authors read, made contributions and approved the final manuscript.
